# Optimization of a Nucleophilic Two-Step Radiosynthesis of 6-*O*-(2-[^18^F]fluoroethyl)-6-*O*-desmethyl-diprenorphine ([^18^F]FE-DPN) for PET Imaging of Brain Opioid Receptors

**DOI:** 10.3390/ijms241713152

**Published:** 2023-08-24

**Authors:** Enikő Németh, Barbara Gyuricza, Viktória Forgács, Paul Cumming, Gjermund Henriksen, János Marton, Beate Bauer, Pál Mikecz, Anikó Fekete

**Affiliations:** 1 Division of Nuclear Medicine and Translational Imaging, Department of Medical Imaging, Faculty of Medicine, University of Debrecen, Nagyerdei Krt. 98, H-4032 Debrecen, Hungary; nemeth.eniko@med.unideb.hu (E.N.); gyuricza.barbara@med.unideb.hu (B.G.); forgacs.viktoria@med.unideb.hu (V.F.); 2 Department of Nuclear Medicine, Bern University Hospital, Freiburgstraße 18, CH-3010 Bern, Switzerland; paul.cumming@insel.ch; 3 School of Psychology and Counselling, Queensland University of Technology, Brisbane QLD-4059, Australia; 4 Norwegian Medical Cyclotron Centre Ltd., Sognsvannsveien 20, N-0372 Oslo, Norway; gjermund.henriksen@syklotronsenteret.no; 5 Institute of Basic Medical Sciences, University of Oslo, N-0317 Oslo, Norway; 6 Institute of Physics, University of Oslo, Sem Sælands Vei 24, N-0371 Oslo, Norway; 7 ABX Advanced Biochemical Compounds Biomedizinische Forschungsreagenzien GmbH, Heinrich-Glaeser-Strasse 10-14, D-01454 Radeberg, Germany; marton@abx.de (J.M.); beatebauerdd@t-online.de (B.B.)

**Keywords:** 6-*O*-(2-[^18^F]fluoroethyl)-6-*O*-desmethyl-diprenorphine, nucleophilic radiosynthesis, opioid receptors, positron emission tomography, orvinols, 6,14-ethenomorphinans

## Abstract

We have established a method for nucleophilic one-pot, two-step radiosynthesis of the popular opioid receptor radioligand 6-*O*-(2-[^18^F]fluoroethyl)-6-*O*-desmethyl-diprenorphine ([^18^F]FE-DPN) from the novel precursor 6-*O*-(2-tosyloxyethyl)-6-*O*-desmethyl- 3-*O*-trityl-diprenorphine (TE-TDDPN), which we designate as the Henriksen precursor. We undertook an optimization of the synthesis conditions, aiming to enhance the accessibility of [^18^F]FE-DPN for positron emission tomography (PET) studies of μ-opioid receptors. Herein, we report an optimized direct nucleophilic ^18^F-fluorination and the deprotection conditions for a fully automated radiosynthesis of [^18^F]FE-DPN on a modified GE Tracerlab FX FE synthesis panel. Starting from 1–1.5 GBq of [^18^F]fluoride and applying an Oasis Max 1cc cartridge for fluorine-18 trapping with a reduced amount of K_2_CO_3_ (5.06 μmol K^+^ ion), [^18^F]FE-DPN ([^18^F]**11**) was produced with 44.5 ± 10.6 RCY (decay-corrected), high radiochemical purity (>99%), and a molar activity of 32.2 ± 11.8 GBq/μmol in 60–65 min.

## 1. Introduction

Opioid receptors (ORs) are plasma membrane proteins belonging to the G-protein-coupled receptor family (GPCRs) [1], which have wide expression throughout the central nervous system, the periphery, and the immune system, where they mediate diverse effects of endogenous opioid peptides and poppy alkaloids [2]. The ORs consist of three classic types (μ-OR, δ-OR and κ-OR) and an additional nonclassical type known as the opioid-like 1 receptor (nociceptin/orphanin FQ peptide receptor, ORL-1/NOP) [3]. The opioid systems are involved in numerous physiological processes [4] and are implicated in pathophysiological processes such as chronic pain, movement disorders, substance abuse, epilepsy, and schizophrenia [5,6].

**Figure 1:**
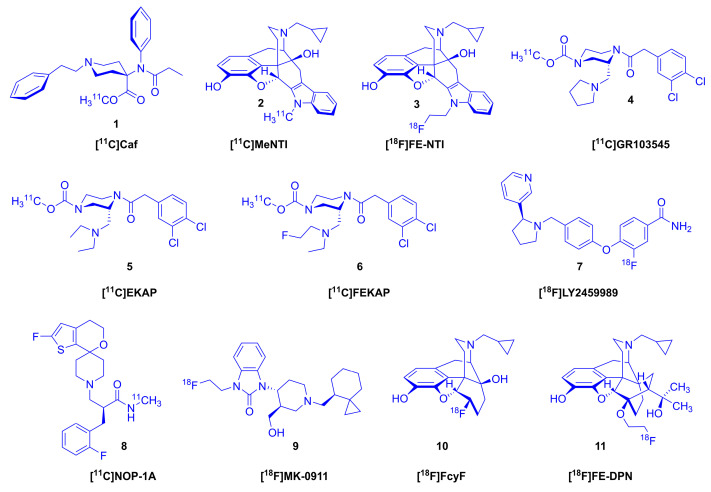
Structures of selected opioid receptor radioligands.

Numerous radiotracers (see Figure 1) targeting ORs have been developed and applied in preclinical research and in clinical investigations to explore the relationships between OR-mediated signaling and (patho)physiology at the molecular level using molecular imaging in vivo with positron emission tomography (PET) [3,7,8,9]. In 1985, the methyl-^11^C ester of carfentanil ([^11^C]carfentanil ([^11^C]Caf, **1**, Figure 1) [10], an agonist ligand highly selective for μ-ORs, was applied in the first human PET investigation of ORs [11]. For studying the δ-ORs, Lever et al. developed the selective antagonists *N*1′-[^11^C]methyl-naltrindole ([^11^C]MeNTI, **2**) [12] and *N*1′-(2-[^18^F]fluoroethyl)-naltrindole ([^18^F]FE-NTI, **3**) [13]. Around the same time, Ravert et al. performed the first radiosynthesis of the κ-OR selective agonist radioligand, [^11^C]GR89696 [14], and its (R)-(-)-enantiomer [^11^C]GR103545 (**4**) [15]. Later Schoultz et al. [16] elaborated an improved method and undertook a PET study in awake rhesus macaques [17]. Recently, following the first human PET investigation with [^11^C]GR103545 (**4**) [18], new κ-OR agonist radiotracers with more advantageous imaging profiles were developed: [^11^C]EKAP (**5**) [19], [^11^C]FEKAP (**6**) [20], and [^18^F]LY2459989 (**7**) [21]. In 2011, the research group of Pike synthesized the NOP antagonist radiotracer [^11^C]NOP-1A (**8**) [22]. Subsequently [^18^F]MK-0911 (**9**) was developed by Hostetler et al. [23].

**Figure 2:**
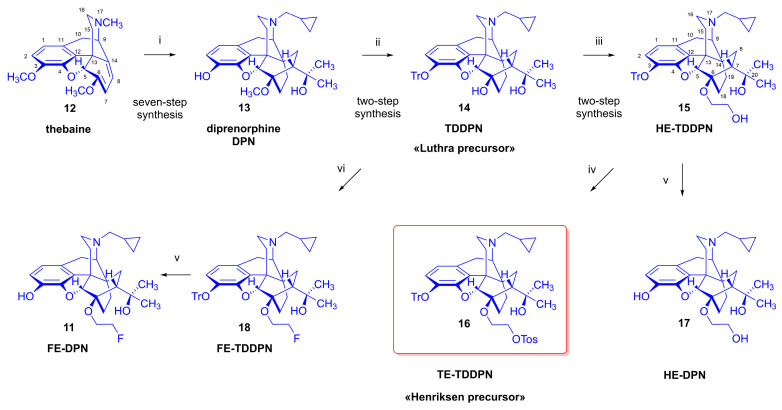
Synthesis of 6-*O*-substituted-diprenorphine derivatives. *Reagents and conditions:* (i): (1) methyl vinyl ketone, reflux, 1 h; (2): H_2_, 10% Pd-C, EtOH, 4 bar, 50 °C, 10 h; (3): methylmagnesium iodide, diethyl ether—benzene, reflux, 2 h; (4): BrCN, CH_2_Cl_2_, RT, 16 h; (5): KOH, diethylene glycol, 165–170 °C, 75 min; (6): cyclopropylmethyl bromide, NaHCO_3_, DMF, 90–95 °C, 20 h; (7): KOH, diethylene glycol, 210–220 °C, nitrogen atmosphere, 2 h; (ii): (1) LiAlH_4_, CCl_4_, THF, reflux, 24 h, (2) trityl chloride, Et_3_N, CH_2_Cl_2_, room temperature, 48 h; (iii): (1) BrCH_2_CH_2_OTBDPS, NaH, DMF, RT, 20 h, (2) 1M Bu_4_NF in THF, RT, 2 h; (iv): Tos_2_O, pyridine, CH_2_Cl_2_, RT, 2 h; (v): acetic acid, H_2_O, 100 °C, 5–10 min, argon atmosphere; (vi): ICH_2_CH_2_F, NaH, DMF, RT, 24 h.

Alongside the development of subtype-selective OR tracers, nonselective ligands have remained in use, notably the μ/κ-OR preferring antagonist *N*^17^-cyclopropylmethyl-6-deoxy-6β-[^18^F]fluoro-noroxymorphone ([^18^F]cyclofoxy, [^18^F]FcyF, **10**, K_i_ (μ-OR) = 2.62 nM, K_i_ (δ-OR) = 89 nM, K_i_ (κ-OR) = 9.3 nM) [24]) which was developed by the research group of Rice [25]. First synthesized by Wester et al. [26], the antagonist ligand 6-*O*-(2-[^18^F]fluoroethyl-6-*O*-desmethyl-diprenorphine ([^18^F]FE-DPN, [^18^F]**11**), showed scant selectivity between OR subtypes in vitro (K_i_ (μ-OR) = 0.24 nM, K_i_ (δ-OR) = 8.0 nM, K_i_ (κ) = 0.2 nM, μ/κ = 1.2, δ/κ = 40). That result was in keeping with earlier findings with [^11^C]diprenorphine ([^11^C]**13**, [^11^C]DPN), a compound first obtained in the 1960s by Bentley et al. through extensive investigations of the Diels–Alder reactions of the poppy alkaloid thebaine (**12**, Figure 2) [27,28,29]. Diprenorphine (**13**), including [^18^F]FE-DPN ([^18^F]**11**), contains a 6,14-*endo*-ethano bridge in ring-C, an *N*^17^-cyclopropylmethyl group, a 7α-((α,α-dimethyl)-methanol) *tertiary* alcohol function, and a phenolic hydroxyl group in position-3. With a comparable affinity in the nanomolar range to all classical ORs in vitro, diprenorphine (**13**) is an antagonist 100 times more potent than *N*^17^-allyl-normorphine [30,31]. Due this high antagonist potency, diprenorphine (**13**) is widely used in veterinary medicine, for waking large animals (deer, horses, elephants, rhinos) that have been immobilized with extremely potent opioid agonists such as carfentanil or etorphine [32], but it is not approved for pharmaceutical use in humans.

[^3^H]DPN ([15,16-^3^H]diprenorphine) was first applied to identify and characterize brain ORs in vitro [33]. In the earliest report of its pharmacological selectivity, Frost et al. [34] found that [^3^H]DPN was μ-OR selective, according to displacement studies in vivo. Subsequently, Jones et al. [35] concluded that the initial attribution of μ-OR selectivity had been due to invalid extrapolation of binding data in vitro to the interpretation of studies ex vivo. Indeed, Raynor et al. [36] found that diprenorphine (**13**) was nearly equipotent in vitro for displacing selective ligands from transformed CHO cells expressing human ORs, i.e., [^3^H]DAMGO (μ-OR, K_i_ = 0.07 nM), [^3^H]naltrindole (δ-OR, K_i_ = 0.23 nM), and [^3^H]U-69,593 (κ-OR, K_i_ = 0.02 nM). Returning to this topic of selectivity of orvinols, recent small animal PET results indicate considerable selectivity of [^18^F]FE-DPN ([^18^F]**11**) for μ-ORs in the brain tissue of living rodents [37], which should favor its use in PET research.

In the 1980s, a research group of the Hammersmith Hospital developed a new type of ^11^C-labeled diprenorphine derivative, namely [*N*^17^-cyclopropylmethyl-^11^C] diprenorphine [38]. The synthesis entailed a reaction of *N*^17^-nordiprenorphine with [carboxy-^11^C]cyclopropanecarbonyl chloride, followed by a reduction of the resultant amide with LiAlH_4_ to the labelled orvinol-type radiotracer. The synthesis of the metabolically more stable [6-*O*-methyl-^11^C]diprenorphine was developed by Lever [39,40] from 3-*O*-*tert*-butyldimethylsilyl-6-*O*-desmethyl-diprenorphine. Subsequently, Luthra et al. [41] introduced a new and more base-stable precursor, 3-*O*-trityl-6-*O*-desmethyl-diprenorphine (TDDPN, **14**) (Figure 2). Following the methodological development work of Lever et al. [40] and Luthra et al. [41], [6-*O*-methyl-^11^C]diprenorphine has been used in numerous PET studies in humans and experimental animals [3].

However, the short physical half-life of carbon-11 (20.34 min) is an impediment to the use of diprenorphine derivatives, as new radiosynthesis is required for each successive PET examination. The longer physical half-life of ^18^F-fluorine (110 min) can thus be logistically advantageous compared to ^11^C-carbon, with respect to longer PET recording times and the logistics of radioligand preparation and delivery to nearby PET centers. Furthermore, favorable nuclear properties of ^18^F-fluorine (97% β+ decay and 635 keV positron energy) enable higher resolution imaging.

Herein, we described a detailed optimized radiosynthetic approach for the preparation of [^18^F]FE-DPN ([^18^F]**11**) applying direct aliphatic ^18^F-fluorination on a GE Tracerlab FX FE automated synthesis module. This convenient radiolabeling method should ultimately enhance the accessibility of [^18^F]FE-DPN ([^18^F]**11**)for PET investigations of µ-ORs.

## 2. Results and Discussion

Tracer molecules can be radiofluorinated with direct or indirect methods [42]. In the former approach, [^18^F]fluoride directly reacts with the precursor designed for radiolabeling though the incorporation of a favorable leaving group. In such applications, the radiolabeling step is followed by the removal of the protective group and the subsequent purification of the desired labeled product [43]. Indirect methods require the previous radiolabeling of a prosthetic group, which is then conjugated to the precursor biomolecule. For this purpose, ^18^F-labeled prosthetic groups are prepared in one or two steps, usually followed by purification, which further increases the time required for complete radiosynthesis [44,45]. Therefore, direct radiofluorination is generally preferable to the indirect method.

**Figure 3:**
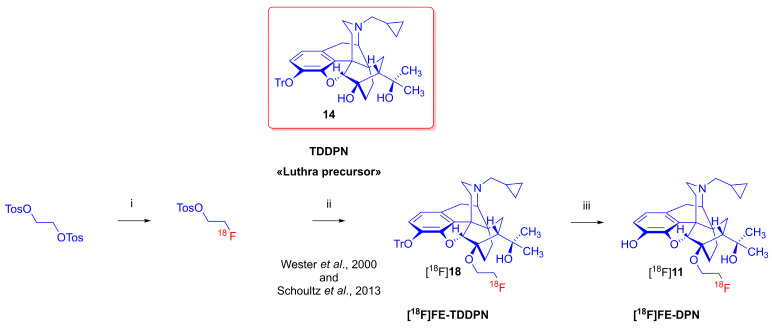
Radiosynthesis of 6-*O*-(2-[^18^F]fluoroethyl)-6-*O*-demethyl-diprenorphine ([^18^F]FE-DPN, ([^18^F]**11**) via indirect radiofluorination. *Reagents and conditions*: (i): [K^+^ ⸦K_222_]^18^F^-^, CH_3_CN, 100 °C, 10 min; (ii): NaH, 100 °C, 10 min; (iii): 2 M HCl, EtOH, 40 °C, 5 min [26,43].

With the limitations of carbon-11 in mind, Wester et al. developed in 2000 a diprenorphine (**13**) derivative labeled with fluorine-18, namely 6-*O*-(2-[^18^F]fluoroethyl)-6-*O*-desmethyl-diprenorphine ([^18^F]FE-DPN, [^18^F]**11**) [26]. The first radiosynthesis of [^18^F]FE-DPN ([^18^F]**11**) utilized the 3-*O*-trityl protected precursor, 3-*O*-trityl-6-*O*-desmethyl-diprenorphine (**14**, TDDPN, «Luthra precursor» [41]), in an indirect, two-pot, three-step procedure (Figure 3). The precursor (**14**) was reacted with the previously radiolabeled prosthetic compound 2-[^18^F]fluoroethyl tosylate ([^18^F]FEOTos) in *N*,*N*-dimethyl formamide in the presence of the strong base sodium hydride. Subsequently, the protecting group was removed through a treatment with 2 M hydrochloric acid, giving [^18^F]FE-DPN ([^18^F]**11**) with molar activity around 37 GBq/μmol in a radiochemical yield of 22 ± 7% at the end of synthesis.

In 2013, Schoultz et al. [46] reported a modified version of the aforementioned indirect radiosynthesis of [^18^F]FE-DPN ([^18^F]**11**) from TDDPN **(14**). Their improved method for the production of [^18^F]FEOTos enabled automated [^18^F]fluoroalkylation on a HBIII module (Scintomics), yielding [^18^F]**11** in >99% chemical and radiochemical purity, radiochemical yield of 25 ± 7% (n = 30), and molar activity of 50–200 GBq/μmol, depending on the molar activity of the starting [^18^F]fluoride. Schoultz et al. [47] generalized this procedure for two further orvinol type radiotracers: 6-*O*-(2-[^18^F]fluoroethyl)-6-*O*-desmethyl-buprenorphine ([^18^F]FE-BPN) and 6-*O*-(2-[^18^F]fluoroethyl)-6-*O*-desmethyl-phenethyl-orvinol ([^18^F]FE-PEO). The successful utilization of [^18^F]FE-DPN ([^18^F]**11**) in various human PET studies has been reported in detail elsewhere [48,49,50,51,52,53,54,55,56,57,58]. The non-radioactive reference substance 6-*O*-(2-fluoroethyl)-6-*O*-desmethyl-diprenorphine (FE-DPN, **11**) was synthesized via three alternative routes [59] and characterized using NMR spectroscopy [60].

The effective and convenient radiosynthesis of radiopharmaceuticals intended for PET imaging is an important prerequisite for their accessibility. To avoid the disadvantage of indirect radiofluorination of diprenorphine (more synthetic and purification steps), a new precursor, namely 6-*O*-(2-tosyloxyethyl)-6-*O*-desmethyl-3-*O*-trityl-diprenorphine (TE-TDDPN, **16**), was developed from TDDPN (**14**) in three steps by Henriksen et al. [59]. For the synthesis of TE-TDDPN (**16**), TDDPN (**14**) was alkylated with 2-bromoethoxy-*tert*-butyldiphenylsilane (2.3 equiv.) in DMF in the presence of sodium hydride (10 equiv.). In the next step, the TBDPS-protecting group was removed with 1 M Bu_4_NF in THF at room temperature. The primary hydroxyl group of 6-*O*-(2-hydroxyethyl)-6-*O*-desmethyl-3-*O*-trityl-diprenorphine (HE-TDDPN, **15**) was tosylated (Tos_2_O, pyridine, CH_2_Cl_2_), with overall 27% yield of the synthesis from compound **14**.

**Figure 4:**
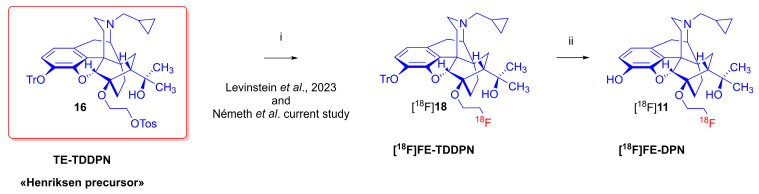
The radiosynthesis of [^18^F]FE-DPN ([^18^F]**11**) via direct radiofluorination. *Reagents and conditions*: (i) [K^+^ ⸦K_222_]^18^F^-^, CH_3_CN, 100 °C, 12 min; (ii): 1 M HCl in 96% EtOH, 80 °C, 12 min (Levinstein et al. 2023) [37].

The availability of this novel TE-TDDPN (**16**) precursor [59] encouraged us to investigate the synthesis of [^18^F]FE-DPN ([^18^F]**11**) through direct radiofluorination (Figure 4). However, in parallel with our research, Levinstein et al. achieved the one-pot, two-step nucleophilic radiosynthesis of [^18^F]FE-DPN ([^18^F]**11**) using an in-house, custom-made radiofluorination module [37]. In their procedure, TE-TDDPN (**16**) was treated with dried [^18^F]fluoride (100 °C, 12 min) followed by a removal of the the trityl protecting group of [^18^F]FE-TDDPN ([^18^F]**18**) with 1 M hydrochloric acid (80 °C, 12 min). The average radiochemical yield was 10% (non-decay corrected), the radiochemical purity was 98%, and the molar activity 2027 GBq/μmol.

Binding studies reported by Levinstein et al. indicated considerable selectivity of [^18^F]FE-DPN ([^18^F]**11**) for μ-ORs [37], in contrast to earlier reports showing ambivalence for the OR-subtype, noted above. An optimized preparation of [^18^F]FE-DPN ([^18^F]**11**) would enable clarification of its subtype-selectivity, as well as generally facilitating PET investigations of ORs. Therefore, we have established an optimized, fully automated procedure for the radiosynthesis and purification of [^18^F]FE-DPN ([^18^F]**11**) using a modified GE TRACERlab FX FE synthesis panel.

**Figure 5:**
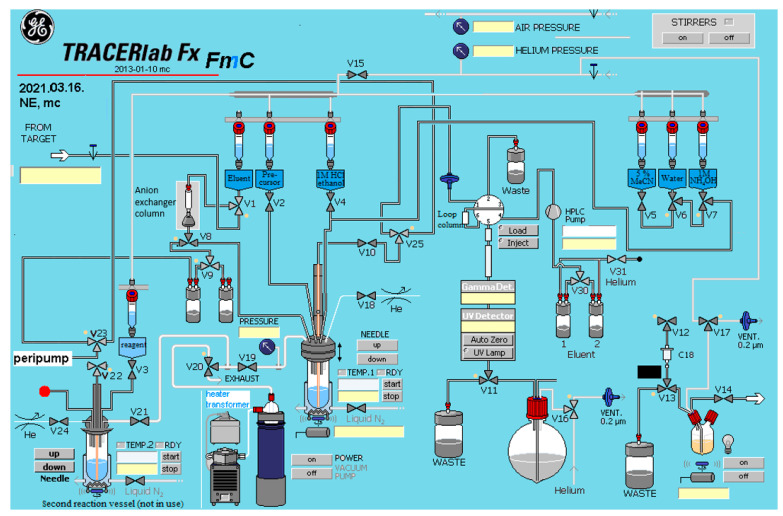
Schematic diagram of the modified TRACERlab FX FE synthesis panel.

The preparation of [^18^F]FE-DPN ([^18^F]**11**) consists of the following steps: fluorine-18 production, [^18^F]fluoride separation, radiolabeling, deprotection, semipreparative HPLC purification, formulation, and quality control. Due to inadequate performance of our initial procedures, we sought to improve the radiochemical yield of the [^18^F]FE-DPN ([^18^F]**11**) synthesis by investigating different labeling conditions, i.e., varying factors such as the amount and type of base used to elute [^18^F]fluoride, precursor concentration, reaction temperature, and reaction time. For the automated radiosyntheses, we used the GE TRA-CERlab FX FE synthesis panel with significant modifications, as presented in Figure 5. While originally designed for electrophile radiosynthesis, we modified it to be suitable for nucleophilic reactions. In particular, we replaced the fluorine–neon inlet with an irradiated water inlet and added a second reactor with several extra valves, which we did not, however, use in these experiments. Moreover, we removed the formulation unit of the panel, in order to have enough reagent vessel for the reaction and the transfer of the reaction mixture for HPLC purification.

The fluorine-18 nuclide (1–1.5 GBq) was produced with a GE PETtrace cyclotron using the ^18^O(p,n)^18^F nuclear reaction. One of the critical steps in the synthesis was the separation of [^18^F]fluoride anion from ^18^O-enriched water. For separating the [^18^F]fluoride ion from ^18^O-enriched water, we tested the following anion exchanger columns: Sep-Pak QMA Plus Light column (130 mg, Waters), SOLA-AX SPE column (10 mg, Thermo Scientific), and Oasis Max 1cc (10 mg, Waters), each with elution mixtures of various compositions.

We first used standard elution conditions for [^18^F]F^-^ ion separation. The anion exchanger column (V8 position) was a Sep-Pak QMA Plus Light column (preconditioned with 2 mL of 14 mg/mL K_2_CO_3_ solution and 10 mL of water), and the elution mixture (V1 position) contained K_2_CO_3_ and Kriptofix 222 (K_222_) as the phase transfer catalyst. The elution solution was a mixture of 120 µL 14 mg/mL aqueous solution of K_2_CO_3_ and 10 mg of K_222_ in acetonitrile (2 mL). However, this resulted in low radiochemical yield and an unidentified radioactive by-product, probably due to the base sensitivity of the precursor and the labeled compound. Previous work indicated 6-*O*-(2-hydroxyethyl)-6-*O*-desmethyl-diprenorphine (**17**, HE-DPN) to be a likely biproduct, which we confirmed by synthesis and structural identification (see in Supplementary Material: Table S1, Figures S1 and S2). The retention time of HE-DPN was 7–8 min in the semi-preparative HPLC chromatogram, while the retention time of [^18^F]FE-DPN was 15 min, thus affording complete separation. During the initial radiosyntheses with relatively large amounts of base, we obtained significant amounts of a radioactive by-product compared to [^18^F]FE-DPN, in a ratio of 3:7 (Supplementary Material Figure S3). We hypothesize that cleavage of the E-ring after nucleophilic ^18^F-fluorination of TE-TDDPN could possibly result in the formation of two radioactive by-products (Supplementary Material Scheme S1).

Thus, it was necessary to reduce the basicity of the reaction mixture to avoid degradation during radiosynthesis. In the subsequent experiments, we applied KHCO_3_ in reduced amounts to the reaction mixture, such that the elution mixture consisted of 120 µL of a 10 mg/mL aqueous KHCO_3_ solution and 5.5 mg of K_222_ in acetonitrile (2 mL). However, the low K^+^ ion concentration resulted in low [^18^F]F^-^ elution efficiency, so we increased the amount of KHCO_3_ to 1.8 mg and the amount of K_222_ to 9 mg, which gave better [^18^F]F^-^ recovery.

In order to further reduce the amount of base and achieve adequate [^18^F]F^-^ elution, we studied other anion exchange columns containing less anion exchange sorbent. We had previously tested the SOLA-AX SPE anion exchange column with 10 mg sorbent for the separation of [^18^F]F^-^ with 80% recovery using a manual loading and elution procedure [61]. Based upon these results, we tested the applicability of the SOLA-AX SPE column (preconditioned with 2 mL of 10% K_2_CO_3_ solution and 10 mL of water) in the automated synthesis apparatus. However, the flow resistance of this column was very low, thus calling for a low-flow loading process for suitable fluorine-18 absorption. This approach proved difficult to implement and was insufficiently robust. Therefore, we decided to abandon using this anion exchange column for the radiosynthesis of [^18^F]FE-DPN ([^18^F]**11**).

Finally, based on the results of Song et al. [62], we began testing the Oasis Max 1cc cartridge (preconditioned with 2 mL of 0.15 M KHCO_3_ solution and 6 mL water) for [^18^F]F^-^ ion separation, which proved to give the best results among the various tested anion exchange columns. For this cartridge, we applied an elution mixture of the following composition: 7 µL of the 50 mg/mL aqueous solution of K_2_CO_3_, 100 µL of K_222_ solution (10 mg/mL in acetonitrile), 100 µL water, and 400 µL acetonitrile. By this method, we succeeded in reducing the ratio of radioactive by-product to product to 6:94 (Supplementary Material Figure S4).

Furthermore, we tested Bu_4_NHCO_3_ as an alternative base for this column, where the elution solution consisted of 50 µL aqueous Bu_4_NHCO_3_ (0.075 M) and 500 µL acetonitrile. This approach gave similar results to using K_2_CO_3_ as a base but with two radioactive by-products in the analytical HPLC chromatogram, in an 8:92 ratio relative to [^18^F]FE-DPN (Supplementary Material Figure S5). After [^18^F]F^-^ elution, the eluate was azeotropically evaporated by heating it at a reduced pressure. We present the [^18^F]fluoride recoveries from different anion exchange columns and conditions in Table 1.

In the first radiosynthesis, 1 mg of TE-TDDPN precursor (**16**) (V2 position) was reacted in 1 mL of dry acetonitrile at 90 °C for 5 min, which failed to produce an adequate incorporation of [^18^F]F^-^. Subsequently, we added, 2 mg TE-DPN precursor in 500 µL dry acetonitrile to the dried [^18^F]fluoride in the reactor and heated the reaction mixture at 100 °C for 10 min. For deprotection, the reactor was cooled to 40 °C. In all subsequent experiments, we used these reaction conditions for the radiosynthesis.

First, we preformed deprotection of the labeled compound with 1 mL of 1M HCl in 96% ethanol (V4 position) at 40 °C for 10 min. However, the reaction time of 10 min proved insufficient for the complete hydrolysis of the trityl group. Consequently, we increased the reaction time to 15 min, while holding the temperature at 40 °C. After deprotection, the reaction mixture was neutralized with 1100 µL of 1M NH_4_OH solution (V7 position).

**Figure 6:**
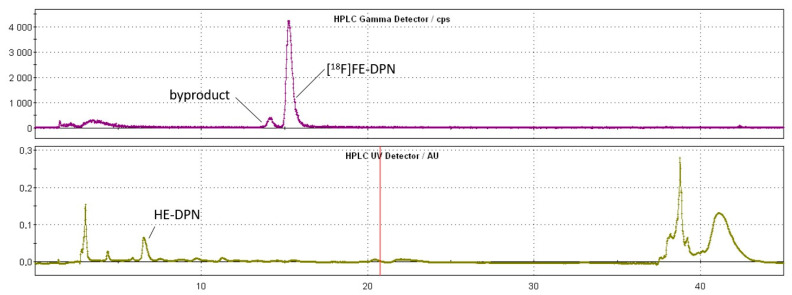
Semipreparative HPLC chromatograms.

For the purification of [^18^F]FE-DPN ([^18^F]**11**) with semipreparative RP-HPLC, the neutralized reaction mixture was diluted with 15 mL water (V6 position). Here, the HPLC retention time of the labeled compound depended on the pH of the eluent and of the sample solution; any substantial deviation from pH 7 shifted the retention time by several minutes. To eliminate this retention time drift, we replaced the semipreparative HPLC injection loop with a home-made reversed-phase pre-column packed with the C18 sorbent from a Sep-Pak C18 Plus Light cartridge. To prevent gas bubbles on the column, we installed a vented filter on the precolumn. Initially, we rinsed the reactor with 10 mL water and transferred this solution to the C18 precolumn. Later, we applied a 5% acetonitrile aqueous solution for reactor washing because the filter otherwise became clogged, probably due to the presence of water-insoluble substances. The final product was purified on a semipreparative HPLC (retention time ~15.5 min) and then formulated. Figure 6 shows the semi-preparative HPLC chromatograms and Table 2 presents the results of the optimization experiments.

**Figure 7:**
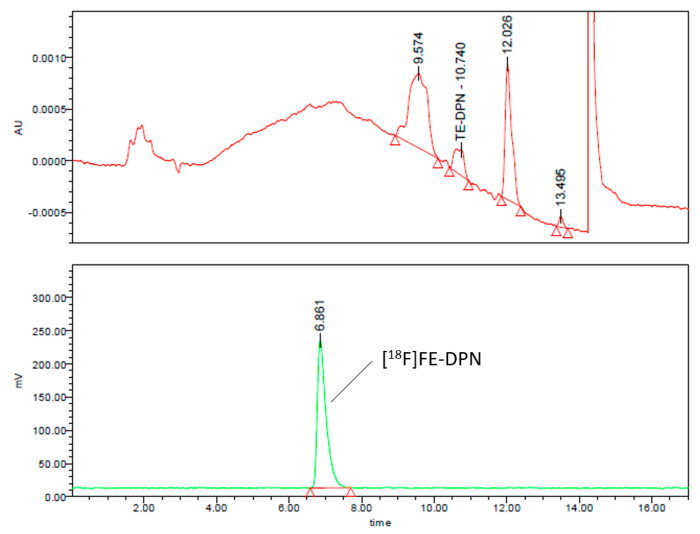
Analytical UV and radio-HPLC chromatograms of the [^18^F]FE-DPN ([^18^F]**11**) using the gradient elution.

For the quality control of [^18^F]FE-DPN ([^18^F]**11**), we developed two analytical HPLC methods. First, we measured the radiochemical purity of the purified product by gradient elution according to the following parameters: Nucleodur C18 Pyramid 5 µm (250 × 4.6 mm) RP column with eluent A: acetonitrile and eluent B: 0.1 M ammonium formate (pH 6.67). The linear gradient was 0 min: 40% A and 60% B; 8 min: 100% A; 11 min: 100% A; 11.1 min: 40% A and 60% B, at a flow rate of 1 mL/min and a UV recording at 284.5 nm. Figure 7 shows the analytical HPLC chromatograms of the product using this gradient elution.

**Figure 8:**
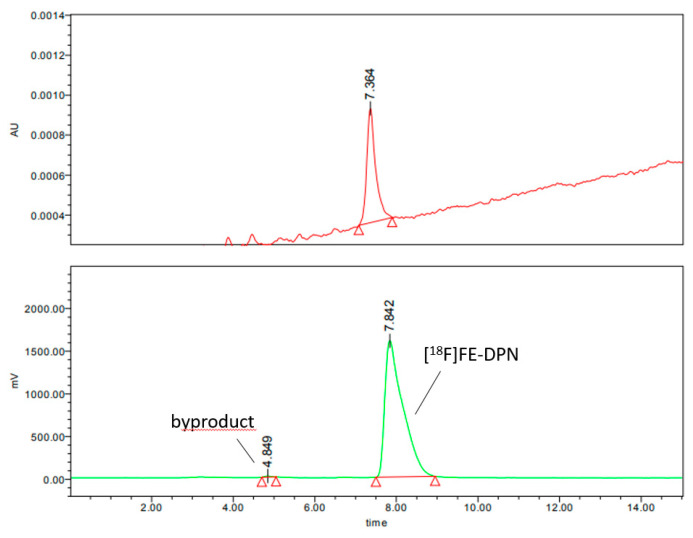
The analytical UV and radio-HPLC chromatograms of the [^18^F]FE-DPN ([^18^F]**11**) using the isocratic HPLC method (radiochemical yield 99.41%).

Second, we developed an isocratic radio-HPLC method using the same column as in the above gradient method. Chromatographic separation was achieved in 15 min using the following solvent formulation: acetonitrile (50%) and 0.1 M ammonium formate, pH 6.67 (50%), with the flow rate at 1 mL/min and the UV detection at 284.5 nm. Figure 8 shows the analytical HPLC chromatograms of the [^18^F]FE-DPN ([^18^F]**11**) using this isocratic HPLC method.

**Figure 9:**
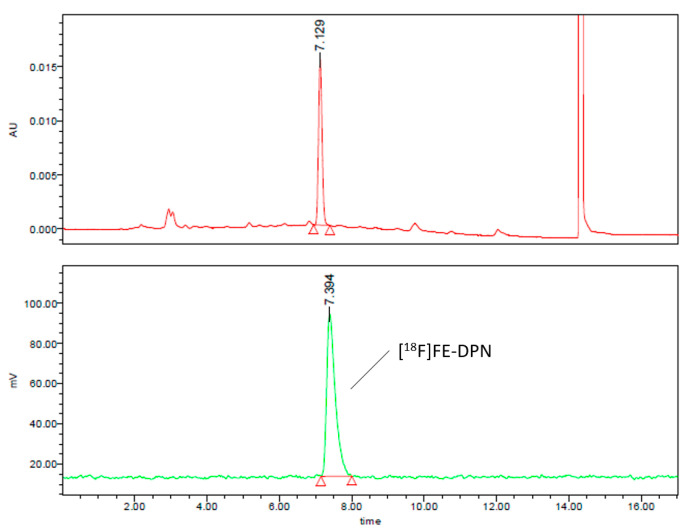
The analytical UV and radio-HPLC chromatograms of the product co-injected with [^19^F]FE-DPN ([^19^F]**11**) standard using the gradient HPLC method.

The chemical identity of the radioactive product was confirmed via a co-injection of the [^19^F]FE-DPN ([^19^F]**11**) standard (Figure 9).

## 3. Conclusions

We successfully optimized the radiosynthesis of the µOR PET ligand [^18^F]FE-DPN ([^18^F]**11**) through direct aliphatic nucleophilic substitution (S_N_2) with [^18^F]fluoride under mild basic conditions. Since [^18^F]FE-DPN was found to be base-sensitive, we investigated the effect of reducing the K^+^ ion concentration on the radiochemical yield. First, we tested the recovery of [^18^F]fluoride at reduced K^+^ ion concentrations from various anion exchange columns, namely Sep-Pak QMA Plus Light, SOLA-AX SPE, and Oasis Max 1cc columns. For the Oasis Max 1cc column, the recovery of [^18^F]fluoride was 92.0 ± 3.7% at 5.06 μmol K^+^ ions, which was the lowest tested effective amount. Furthermore, starting from 1 to 1.5 GBq of [^18^F]fluoride and applying the Oasis Max 1cc anion-exchange cartridge for fluorine-18 trapping with 5.06 μmol K^+^ ions, [^18^F]FE-DPN was prepared with 44.5 ± 10.6 RCY (decay-corrected), high radiochemical purity (>99%), and a relatively high molar activity of 32.2 ± 11.8 GBq/μmol for 60–65 min with a modified GE TRACERlab FX FE synthesis panel. With this procedure, the radiochemical yield of [^18^F]FE-DPN radiosynthesis increased significantly while the reaction time decreased compared to previously published methods [26,37,47].

In addition, with this low K^+^ ion concentration method, we successfully reduced the amount of radioactive by-products in the reaction mixture. During quality control, the by-product HE-DPN (17) was not detectable on the analytical UV HPLC chromatogram of the [^18^F]FE-DPN ([^18^F]**11**) product, nor were any other UV-active impurities present. Tetrabutylammonium bicarbonate was also tested as a phase transfer catalyst for the Oasis Max 1cc column and gave good radiochemical yields but with the formation of new, unidentified impurities.

The results of our optimization experiments enable radiochemists to efficiently and routinely produce the [^18^F]FE-DPN ([^18^F]11) radiotracer for μOR PET imaging in research and clinical trials. In addition, based on our results, the application of Oasis Max 1cc anion-exchange cartridge for [^18^F]F^-^ ion separation can make the radiosynthesis of other base-sensitive radioactive tracers more efficient by using a reduced amount of potassium ions.

## 4. Methods and Materials

All commercial chemicals were obtained from Sigma-Aldrich and were used without further purification. The 6-*O*-(2-tosyloxyethyl)-6-*O*-desmethyl-3-*O*-trityl-diprenorphine (TE-TDDPN, **16**) for [^18^F]FE-DPN radiosynthesis ([^18^F]**11**) and standard [^19^F]FE-DPN ([^19^F]**11**) were provided by ABX advanced biochemical compounds Biomedizinische Forschungsreagenzien GmbH (Radeberg, Germany). [^18^O]H_2_O was purchased from Rotem Industries Ltd. (Mishor Yamin D.N Arava, Israel). [^18^F]Fluoride was produced by a GE PETtrace cyclotron at Division of Nuclear Medicine, Department of Medical Imaging, University of Debrecen, Debrecen, Hungary. Sep-Pak QMA Plus Light (130 mg, WAT023525), Oasis MAX 1 cc (10 mg, 186006341), and Sep-Pak C18 Plus Light (130 mg, WAT023501) cartridges were purchased from Waters Corp. (Milford, MA, USA). SOLA-AX SPE cartridges (10 mg, TF60109-003) were purchased from Thermo Scientific (Minneapolis, MN, USA). All radiosyntheses were performed in the TracerLab FX FE (GE Healthcare, Madison, WI, USA) module. HPLC-grade solvents were used for HPLC (J.T. Baker, Phillipsburg, New Jersey, USA) after membrane filtering (0.45 µm, Whatman membrane filter). All radioactivities were measured with a CAPINTEC CRC-15PET dose calibrator. The semipreparative RP-HPLC was carried out with Sykam S 1021 isocratic HPLC pump connected with Knauer UV and Geiger–Müller tube detectors using Nucleodur C18 Pyramid 5 µm (250 × 10 mm) column with eluent: 65% 0.1 M ammonium-formate, 35% acetonitrile (pH = 7). Analytical radio-HPLC was performed on a Waters 2695 Alliance HPLC system using UV and CsI scintillation detectors (ATOMKI, Debrecen, Hungary). A Nucleodur C18 Pyramid 5 µm (250 × 4.6 mm) column was applied with the following solvent system: A: acetonitrile; B: ammonium-formate (1 M; pH 6.67). Deionized water (Milli-Q, 18.2 MΩcm^−1^, Merck, Kenilworth, NJ, USA) was used for HPLC analysis.

### 4.1. [^18^F]fluoride Production and Separation

No-carrier added (NCA) [^18^F]fluoride was produced through the ^18^O(p,n)^18^F nuclear reaction, and 3.5 mL of ^18^O-enriched water (>95% [^18^O]H_2_O, Rotem Industries Ltd., Mishor Yamin D.N, Arava, Israel) was irradiated with a beam of protons (16.5 MeV, 70 µA) from a GE PETtrace cyclotron. For these experiments the target was rinsed with deionized water, yielding 1–3 GBq [^18^F]fluoride, which was separated from the aqueous solution using an anion exchange column. When using the Sep-Pak QMA Plus Light cartridge, the following elution mixtures were used: **A** eluent: 120 µL of the 14 mg/mL aqueous solution of K_2_CO_3_, 10 mg of K_222_ solution in acetonitrile (2 mL); **B** eluent: 120 µL of the 10 mg/mL aqueous solution of KHCO_3_ and 5.5 mg of K_222_ solution in acetonitrile (2 mL); **C** eluent: 180 µL of the 10 mg/mL aqueous solution of KHCO_3_ and 9 mg of K_222_ solution in acetonitrile (2 mL). When using the Oasis Max 1cc cartridge, the following elution mixtures were used: **D** eluent: 7 µL of the 50 mg/mL aqueous solution of K_2_CO_3_, 100 µL of K_222_ solution (10 mg/mL in acetonitrile), 100 µL water and 400 µL acetonitrile; **E** eluent: 50 µL aqueous Bu_4_NHCO_3_ (0.075 M) and 500 µL acetonitrile. In all cases, the eluate was evaporated in several steps with a flow of helium under reduced pressure (20 kPa), first at 70 °C for 70 s, followed by 105 °C for 75 s, and finally at 120 °C for 290 s. In the last step, the pressure was reduced to 5 kPa, and the reactor was cooled to 60 °C.

### 4.2. Radiosynthesis of [^18^F]FE-DPN ([^18^F]11)

Furthermore, 2 mg TE-TDDPN (**16**) precursor in 500 µL dry acetonitrile was added to the dried [^18^F]fluoride and heated at 100 °C for 10 min. Then, the reactor was cooled to 40 °C and the reaction mixture was treated with 1 mL of 1 M HCl in 96% ethanol at 40 °C for 15 min and finally neutralized with 1100 µL of 1M NH_4_OH solution.

### 4.3. Purification of [^18^F]FE-DPN ([^18^F]11)

For semipreparative RP-HPLC purification, the neutralized reaction mixture was diluted with 15 mL water. A home-made stainless-steel reverse-phase pre-column (8 × 22 mm, ID × length) was used as an HPLC injection loop, which was filled with the sorbent taken from a Sep-Pak C18 Plus Light column (Waters) and preconditioned via passage of 10 mL H_2_O, 10 mL 96% EtOH, and 10 mL H_2_O. A vented filter was applied to prevent gas bubbles in the column. First, the diluted reaction mixture was pushed through the C18 precolumn. Then, the reactor was rinsed with 5% acetonitrile aqueous solution, and the eluant was passed through the C18 precolumn. Semipreparative HPLC was performed using a Nucleodur C18 Pyramid 5 µm (250 × 10 mm) RP column. The eluent was a mixture of 65% 0.1 M ammonium formate and 35% acetonitrile (pH 7). First, the adsorbed compounds from the C18 precolumn were delivered at a flow rate of 2 mL/min to the HPLC separation column, and then the purification was carried out at a flow rate of 5 mL/min. For formulation, the fraction containing the [^18^F]FE-DPN ([^18^F]**11**) was diluted with water and then passed through a Sep-Pak C18 Plus Light column (preconditioned with 10 mL H_2_O, 10 mL 96% EtOH, and 10 mL H_2_O), which was washed with 5 mL water and then eluted with 96% ethanol in 100 µL fractions. Usually, the third fraction contained the highest radioactivity (60–80% of the total activity), which was diluted to a final volume of 1 mL with isotonic saline solution.

### 4.4. Quality Control

A gradient and an isocratic analytical HPLC method were developed using Nucleodur C18 Pyramid 5 µm (250 × 4.6 mm) column with eluent A: acetonitrile and eluent B: 0.1 M ammonium formate (pH 6.67). At a constant 1 mL/min flow rate, the linear gradient conditions were 0 min: 40% A and 60% B; 8 min: 100% A; 11 min: 100% A; 11.1 min: 40% A and 60% B. The isocratic method used the following solvent composition: 50% acetonitrile and 50% 0.1 M ammonium formate (pH 6.67).

## Figures and Tables

**Table 1:** The [^18^F]fluoride recovery from different anion exchange columns.

Cartridge	Eluent Composition						Bound [^18^F]F^-^ (%)	Eluted [^18^F]F^-^ (%)	[^18^F]F^-^ Recovery (%)
	Base (mg)			Kryptofix 222 (mg)	Water (µL)	Acetonitrile (µL)			
	K_2_CO_3_ (mg)	KHCO_3_ (mg)	Bu_4_NHCO_3_ (mg)						
Sep-Pak QMA Plus Light	1.68	-	-	10	120	2000	99 ± 1	97.2 ± 2.1	96.2 ± 1.9
	-	1.2	-	5.5	120	500	99	50.5	50.0
	-	1.8	-	9	180	2000	100	97.4	97.4
SOLA-AX SPE (10 mg)	0.35	-	-	1	47	500	48 ± 27	96.8 ± 1.7	46.5 ± 25.3
Oasis Max 1cc (10 mg)	0.35	-	-	1	47	500	97.6 ± 1.6	92.0 ± 3.7	89.8 ± 4.9
	-	-	0.72	-	50	500	99.4 ± 0.3	94.1 ± 1.0	93.5 ± 0.9

**Table 2:** The resulting radiochemical yields and molar activities during different conditions.

Cartridge	K^+^ (µmol)	Precursor (µmol)	K^+^/Precursor Molar Ratio	Radiochemical Yield ** Corrected with Elution Efficiency (%)	Molar Activity (GBq/µmol)
QMA light SepPak	24.31	1.17	20.8	<1 (n = 2)	n.d.
	17.98	2.35	7.7	6.9 (n = 1)	94
	11.99	2.35	5.1	6.3 (n = 1)	4.4
OasisMax 1cc	5.06	2.35	2.2	44.5 ± 10.6 (n = 3)	32.2 ± 11.8
	3.75 *	2.35	1.6 *	40.8 ± 30.3 (n = 2)	20.4 ± 21.5

* Bu_4_N^+^ instead of K^+^. ** Decay corrected.

## Data Availability

Not applicable.

## Supplementary Materials

The following supporting information can be downloaded at https://www.mdpi.com/article/10.3390/ijms241713152/s1.
