# Supplementary material for: Optimization of a Nucleophilic Two-Step Radiosynthesis of 6-O-(2-[18F]fluoroethyl)-6-O-desmethyl-diprenorphine ([18F]FE-DPN) for PET Imaging of Brain Opioid Receptors
Source: Int J Mol Sci. 2023 Aug 24;24(17):13152. doi: 10.3390/ijms241713152 (PMC10487412; doi:10.3390/ijms241713152)
Supplement: Supplementary file 1 [file ijms-24-13152-s001.zip › ijms-2480482-supplementary.pdf]

CONTENTS

		Page
	ABBREVIATIONS	3
1.	EXPERIMENTAL SECTION	4
1.1	General Methods	4
1.2	Chemistry	4
	1.2.1. Synthesis of 6-*O*-(2-Hydroxyethyl)-6-*O*-desmethyl-diprenorphine (HE-DPN, **17**)	4
1.3.	Supplementary references	5
1.4.	NMR assignments	6
	Table S1 ^{1}H and ^{13}C chemical shifts and coupling constants of HE-DPN (**17**)	6
1.5.	SPECTRAL DATA (^{1}H NMR, ^{13}C NMR)	7

FIGURE	SPECTRUM	
**Figure S1**	^{1}H NMR spectrum of HE-DPN (**17**) in $CDCl_3$ + DMSO-d_6	7
**Figure S2**	^{13}C NMR spectrum of HE-DPN (**17**) in $CDCl_3$ + DMSO-d_6	8
**Figure S3**	Semi-preparative HPLC chromatogram of the reaction mixture when C eluent was used as an elution mixture for [^{18}F]F^- separation with Sep-Pak QMA light cartridge	9
**Scheme S1**	Chemical structure of possible by-products	9
**Figure S4**	Semi-preparative HPLC chromatogram of the reaction mixture when D eluent was used as an elution mixture for [^{18}F]F^- separation with Oasis Max 1cc cartridge	10
**Figure S5**	Analytical HPLC chromatogram of the collected by-products when E eluent was used as an elution mixture for [^{18}F]F^- separation with Oasis Max 1cc cartridge	10

Abbreviations

BPN	=	20*S*-buprenorphine (Temgesic, Buprenex, Subutex)
Caf	=	carfentanil; 4-[(1-oxopropyl)phenylamino]-1-(2-phenylethyl)-4-piperidinecarboxylic acid methyl ester
CPM	=	cyclopropylmethyl group
*c*Prop	=	proton/carbon of the cyclopropyl ring
DDPN	=	6-*O*-desmethyl-diprenorphine
DPN	=	diprenorphine (Revivon, M5050)
EKAP	=	(*R*)-methyl 4-(2-(3,4-dichlorophenyl)acetyl)-3-((diethylamino)methyl)piperazine-1-carboxylate
FcyF	=	cyclofoxy; N^{17}-cyclopropylmethyl-6-deoxy-6β-fluoro-noroxymorphone
FE-BPN	=	6-*O*-(2-fluoroethyl)-6-*O*-desmethyl-buprenorphine
FE-DPN	=	6-*O*-(2-fluoroethyl)-6-*O*-desmethyl-diprenorphine
FEKAP	=	(*R*)-methyl 4-(2-(3,4-dichlorophenyl)acetyl)-3-((ethyl(2-fluoroethyl)amino)methyl)piperazine-1-carboxylate
FE-NTI	=	*N*1'-(2-fluoroethyl)-naltrindole; BU97001
FEOTos	=	2-fluoroethyl tosylate
FE-TDDPN	=	6-*O*-(2-fluoroethyl)-6-*O*-desmethyl-3-*O*-trityl-diprenorphine
FE-TDPEO	=	6-*O*-(2-fluoroethyl)-6-*O*-desmethyl-3-*O*-trityl-phenethyl-orvinol
FE-PEO	=	6-*O*-(2-fluoroethyl)-6-*O*-desmethyl-phenethyl-orvinol
GPCRs	=	G-protein coupled receptor family
GR89696	=	4-[(3,4-dichlorophenyl)acetyl]-3-(*R*,*S*)-(1-pyrrolidinylmethyl)-1-piperazine carboxylic acid methyl ester
GR103545	=	4-[(3,4-dichlorophenyl)acetyl]-3-(*R*)-(1-pyrrolidinylmethyl)-1-piperazine carboxylic acid methyl ester
HE-DPN	=	6-*O*-(2-hydroxyethyl)-6-*O*-desmethyl-diprenorphine
HE-TDDPN	=	6-*O*-(2-hydroxyethyl)-6-*O*-desmethyl-3-*O*-trityl-diprenorphine
L-Selectride	=	lithium tri-*sec*-butylborohydride
LY2459989	=	3-fluoro-4-[4[[(2*S*)-2-(3-pyridyl)pyrrolidin-1-yl]methyl]phenoxy] benzamide
MeNTI	=	*N*1'-methy-naltrindole; methyl-naltrindole
MK-0911	=	1-(2-fluoroethyl)-3-[(3*R*,4*R*)-3-hydroxymethyl)-1-[[(8*S*)-spiro[2.5]octan-8-yl]methyl]piperidin-4-yl]benzimidazol-2-one
NOP	=	opioid-like 1 receptor; ORL-1; nociceptin/orphanin FQ peptide receptor
NOP-1A	=	(2*S*)-2-[(2-fluorophenyl)methyl]-3-[2-fluorospiro[4,5-dihydro-thieno[2,3-c]pyran-7,4-piperidine]-1'-yl)-*N*-methyl-propanamide
NTI	=	naltrexone-indole; naltrindole
δ-OR	=	delta opioid receptor
κ-OR	=	kappa opioid receptor
µ-OR	=	mu opioid receptor
PET	=	positron emission tomography
RT	=	room temperature
TBDPS	=	*tert*-butyldiphenylsilyl group
TE-TDDPN	=	6-*O*-(2-tosyloxyethyl)-6-*O*-desmethyl-3-*O*-trityl-diprenorphine
TDDPN	=	3-*O*-trityl-6-*O*-desmethyl-diprenorphine
Tr	=	trityl group = triphenylmethyl group
TrCl	=	trityl chloride

1. EXPERIMENTAL SECTION

1.1 General Methods

Reagents and solvents were obtained from commercial suppliers and were used without further purification. Column chromatography was performed on a Kieselgel 60 Merck 1.09385 (0.040-0.063 mm) column. Analytical TLC was accomplished on Macherey-Nagel Alugram® Sil G/UV_{254} 40x80 mm aluminum sheets [0.25 mm silica gel with fluorescent indicator] with the following eluent systems (each (v/v)): [A]: dichloromethane-methanol 95:5, [B]: dichlormethane-methanol 9:1. The spots were visualized with a 254 nm UV lamp or with 5 % phosphomolybdic acid in ethanol.

NMR spectra: All the NMR experiments were recorded on a Bruker AV 500 (Avance 500 MHz) spectrometer operating at 298 K, using BBO probehead (hp workstation xw 5000, software: Bruker TOPSPIN 1.3). For the ^{1}H-NMR experiment, 10 mg of HE-DPN (**17**) was dissolved in 500 µL of deuterated chloroform ($CDCl_3$). For measuring the ^{13}C-NMR- and 2D-spectra: 20 mg sample of HE-DPN (**17**) was dissolved in 500 µL $CDCl_3$ (for complete dissolution of the sample, additionally one drop of DMSO-d_6 was given). Chemical shifts (δ) are reported in parts per million (ppm), and coupling constants (J) reported in Hertz. ^{1}H and ^{13}C NMR chemical shifts were referenced to the residual peak of $CDCl_3$ at δ 7.26 (^{1}H) and 77.16 ppm (^{13}C), for proton and carbon, respectively. The conditions of the spectra recording were as follows: ^{1}H-NMR spectra: observation frequency: 500.130 MHz; for the ^{13}C-NMR spectra: observation frequency: 125.758 MHz,

1.2 Chemistry

The precursor molecule TE-TDDPN (**5**) was synthesized starting from 3-*O*-trityl-6-*O*-desmethyl-diprenorphine (**14**, TDDPN, [1]) according to literature procedures [2].

1.2.1. Synthesis of 6-*O*-(2-hydroxyetyl)-6-*O*-desmethyl-diprenorphine (17, HE-DPN)

(5R,6R,7R,9R,13S,14S)-(5α,7α)-17-Cyclopropylmethyl-4,5-epoxy-18,19-dihydro-6-(2-hydroxyethoxy)-3-hydroxy-α,α-dimethyl-6,14-ethenomorphinan-7-methanol (**17**, HE-DPN, reference standard for potential byproduct of the [^{18}F]FE-DPN synthesis)

HE-TDDPN (**15**, 100 mg, 0.14 mmol) was dissolved in a mixture of acetic acid (11 mL) and water (3 mL). The solution was stirred at 100 °C for ten minutes under argon. Analytical TLC of the product mixture after ten minutes reaction time showed absence of starting material. The solution was allowed to cool to room temperature and then poured into ice water (50 mL). The pH of the mixture was adjusted to 9 with 25 m/m % aqueous ammonia solution (ca. 20 mL). The suspension was extracted with a 9:1 (v/v) mixture of dichloromethane-methanol (3 × 50 mL). The combined organic phase was extracted with brine, dried (Na_2SO_4) and the solvent evaporated. The residue (ca. 130 mg) was purified by column chromatography on silica gel (Kieselgel 10g, fractions: 10 mL, eluent system: [A]). Yield: 61 mg (93%), m.p. 230-245 °C (decomposition), TLC: R_f [A] = 0.14, R_f [B] = 0.48. ^{1}H NMR ($CDCl_3$ + DMSO-d_6): δ = 0.02 (m, 2H, cPropCH_{2syn}), 0.38 (m, 2H, cPropCH_{2anti}), 0.65 (tt, J = 12.6 Hz, 3.6 Hz, 1H, 19-H_{syn}), 0.69 (m, 1H, cProp*CH*), 0.93 (m, 1H, 19-H_{anti}), 0.97 (dd, $^{2}J_{8\alpha,8\beta}$ = 13.1 Hz, $^{3}J_{8\alpha,7\beta}$ = 9.4 Hz, 1H, 8α-H), 1.09 (s, 3H, 20-CH_3), 1.30 (s, 3H, 20-CH_3), 1.55 (dd, $^{2}J_{15eq,15ax}$ = 13.1 Hz, $^{3}J_{15eq,16ax}$ = 2.3 Hz, 1H, 15-H_{eq}), 1.65 (m, 1H, 18-H_{syn}), 1.72 (m, 1H, 18-H_{anti}), 1.84 (app t, $^{3}J_{7\beta,8\alpha}$ = 9.6 Hz, 1H, 7β-H), 1.92 (m, 1H, 15-H_{ax}), 2.09 (dd, $^{2}J_{10\alpha,10\beta}$ = 18.5 Hz, $^{3}J_{10\alpha,9\alpha}$ = 6.3 Hz, 1H, 10α-H), 2.14-2.20 (m, 2H, NCH_2 (a) and 16-H_{ax}), 2.26 (dd, J_{BA} = 12.7 Hz, J_{BX} = 5.7 Hz, 1H, NCH_2 (b)), 2.51 (dd, $^{2}J_{16eq,16ax}$ = 12.0 Hz, $^{3}J_{16eq,15ax}$ = 5.0 Hz, 1H, 16-H_{eq}), 2.75 (ddd, $^{2}J_{8\beta,8\alpha}$ = 13.8 Hz, $^{3}J_{8\beta,7\beta}$ = 12.4 Hz, $^{4}J_{8\beta,19syn}$ = 3.6 Hz, 1H, 8β-H), 2.86

(d, $^2J_{10\beta,10\alpha}$ = 18.5 Hz, 1H, 10β-H), 2.89 (d, $^3J_{9\alpha,10\alpha}$ = 6.3 Hz, 1H, 9α-H), 3.17 (br s, 1H, 6-O-CH_2CH_2*OH*), 3.60-3.63(m, 2H, 6-O-*CH_2*CH_2OH), 3.67-4.03 (m, 2H, 6-O-CH_2*CH_2*OH), 4.29 (d, 1H, $^2J_{5\beta,18anti}$ = 1.7 Hz, 5β-H), 5.07 (br s, 1H, 20-OH), 6.36 (d, $^3J_{1,2}$ = 8.0 Hz, 1H, 1-H), 6.56 (d, $^3J_{2,1}$ = 8.0 Hz, 1H, 2-H), 7.92 (s, 1H, 3-*OH*). ^{13}C NMR ($CDCl_3$ + DMSO-*d_6*): δ = 3.0 (cProp(a)), 3.9 (cProp(b)), 9.1 (cProp*CH*), 18.1 (C-18), 22.3 (C-10), 24.7 (20-*CH_3*), 29.6 (20-*CH_3*), 29.7 (C-19), 32.1 (C-8), 35.3 and 35.7 (C-14 and C-15), 43.5 (C-16), 46.7 and 47.7 (C-13 and C-7), 57.9 (C-9), 59.6 (N*CH_2*), 61.9 (6-OCH_2*CH_2*OH), 65.6 (6-O*CH_2*CH_2OH), 74.1 (C-20), 80.2 (C-6), 96.6 (C-5), 116.4 (C-2), 119.0 (C-1), 126.9 (C-11), 132.1 (C-13), 138.0 (C-3), 145.5 (C-4), $C_{27}H_{37}NO5$ (455,60).

1.3. Supplementary references

[1] Luthra, S. K.; Brady, F.; Turton, D. R.; Brown, D. J.; Dowsett, K.; Waters, S. L.; Jones, A. K. P.; Matthews, R. W.; Crowder, J. C., Automated radiosyntheses of [6-*O*-methyl-^{11}C]diprenorphine and [6-*O*-methyl-^{11}C]buprenorphine from 3-*O*-trityl protected precursors. *Appl. Radiat. Isot.* **1994**, *45*, 857-873.

[2] Marton, J.; Cumming, P.; Bauer, B.; Henriksen, G., A new precursor for the radiosynthesis of 6-*O*-(2- [^{18}F]fluoroethyl)-6-*O*-desmethyl-diprenorphine ([^{18}F]FE-DPN) by nucleophilic radiofluorination. *Lett. Org. Chem.* **2021**, *18*, 5, 344-352.

1.4. NMR assignments

TABLE S1 ^{1}H and ^{13}C NMR chemical shifts and coupling constants of HE-DPN (**17**)

**Position**	**HE-DPN (17)** δ ^{13}C	^{1}H (m, *J* in Hz)
1	119.0	6.36 (d, 8.0)
2	116.4	6.56 (d, 8.0)
3	138.0	-
4	145.5	-
5β	96.6	4.29 (d, 1.7)
6	80.2	-
7β	47.7	1.84 (app t, 9.6)
8α	32.1	0.97 (dd, 13.1, 9.4)
8β		2.75 (ddd, 13.8, 12.4, 3.6)
9α	57.9	2.89 (d, 6.3)
10α	22.3	2.09 (dd, 18.5, 6.3)
10β		2.86 (d, 18.5)
11	126.9	-
12	132.1	-
13	46.7	-
14	35.7	-
15_{ax}	35.3	1.92 (m)
15_{eq}		1.55 dd (13.1, 2.3)
16_{ax}	43.5	2.14-2.20 (m) overlapped by the signals of NCH_2 (a)
16_{eq}		2.51 (dd, 12.0, 5.0)
18_{syn}	18.1	1.65 (m)
18_{anti}		1.72 (m)
19_{syn}	29.7	0.65 (tt, 12.6, 3.6)
19_{anti}		0.93 (m)
20	74.1	-
**Others**		
20-CH_3	24.7	1.09 (s)
20-CH_3	29.6	1.30 (s)
cPropCH_{2syn}	3.0	0.02 (m)
cPropCH_{2anti}	3.9	0.38 (m)
cProp*CH*	9.1	0.69 (m)
NCH_2 (a)	59.6	2.14-2.20 (m, overlapped by the signals of 16-H_{ax})
NCH_2 (b)		2.26 (dd, 12.7, 5.7)
CH_2CH₂OH	65.6	3.60-3.63 (m)
CH₂CH_2OH	61.9	3.67-4.03 (m)
CH₂CH₂*OH*		3.17 (br s)
3-*OH*	-	7.92 (br s)
20-*OH*	-	5.07 (br s)

a: observation frequency: 500.130 MHz (for ^{1}H-NMR), 125.758 MHz (for ^{13}C-NMR), δ in ppm; in $CDCl_3$ + DMSO-d_6

1.5. SPECTRAL DATA (^{1}H NMR, ^{13}C NMR)

Figure S1 ^{1}H NMR spectrum of HE-DPN (**17**) in $CDCl_3$ + DMSO-d_6

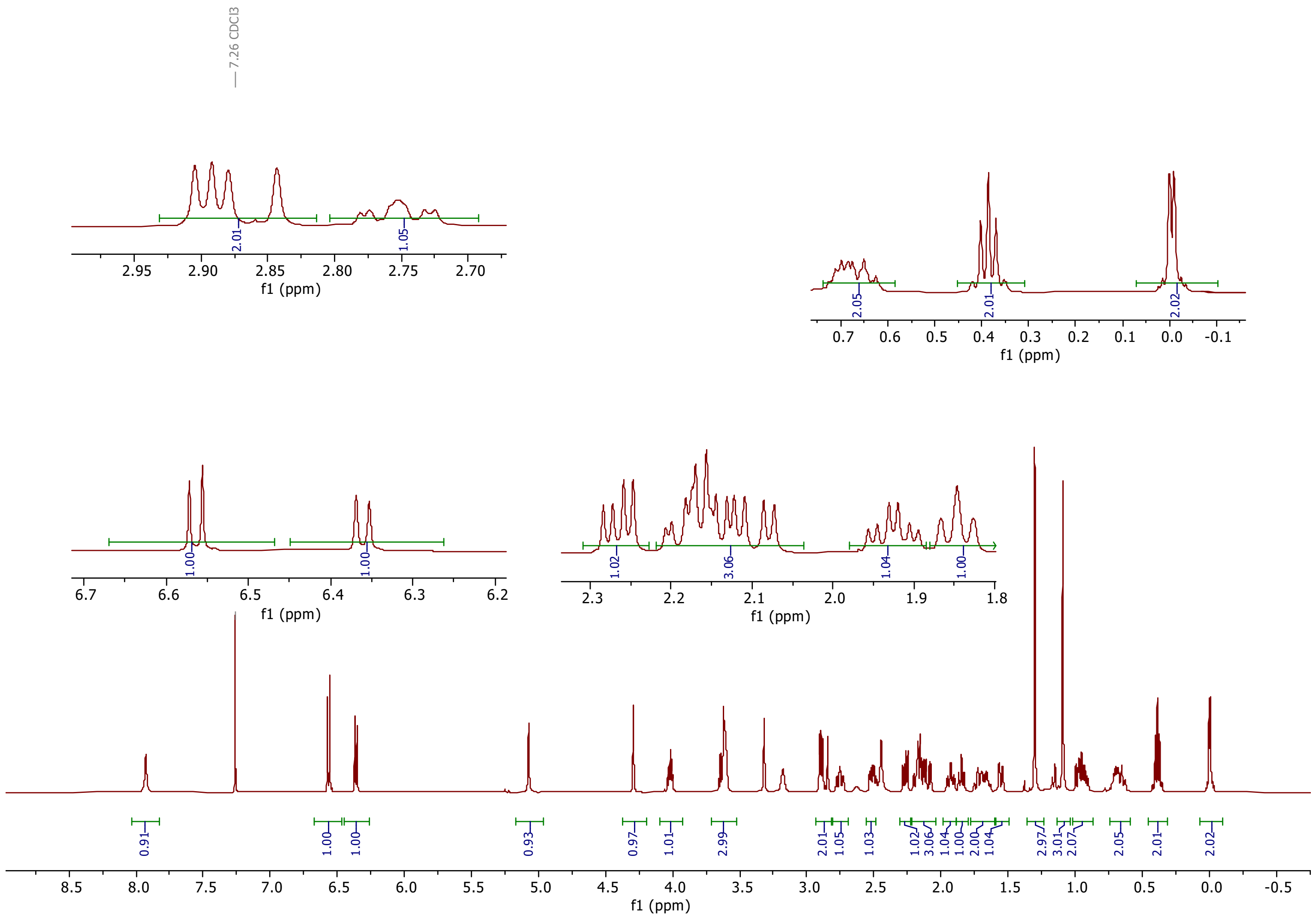

Figure S2 ^{13}C NMR spectrum of HE-DPN (**17**) in $CDCl_3$ + DMSO-d_6

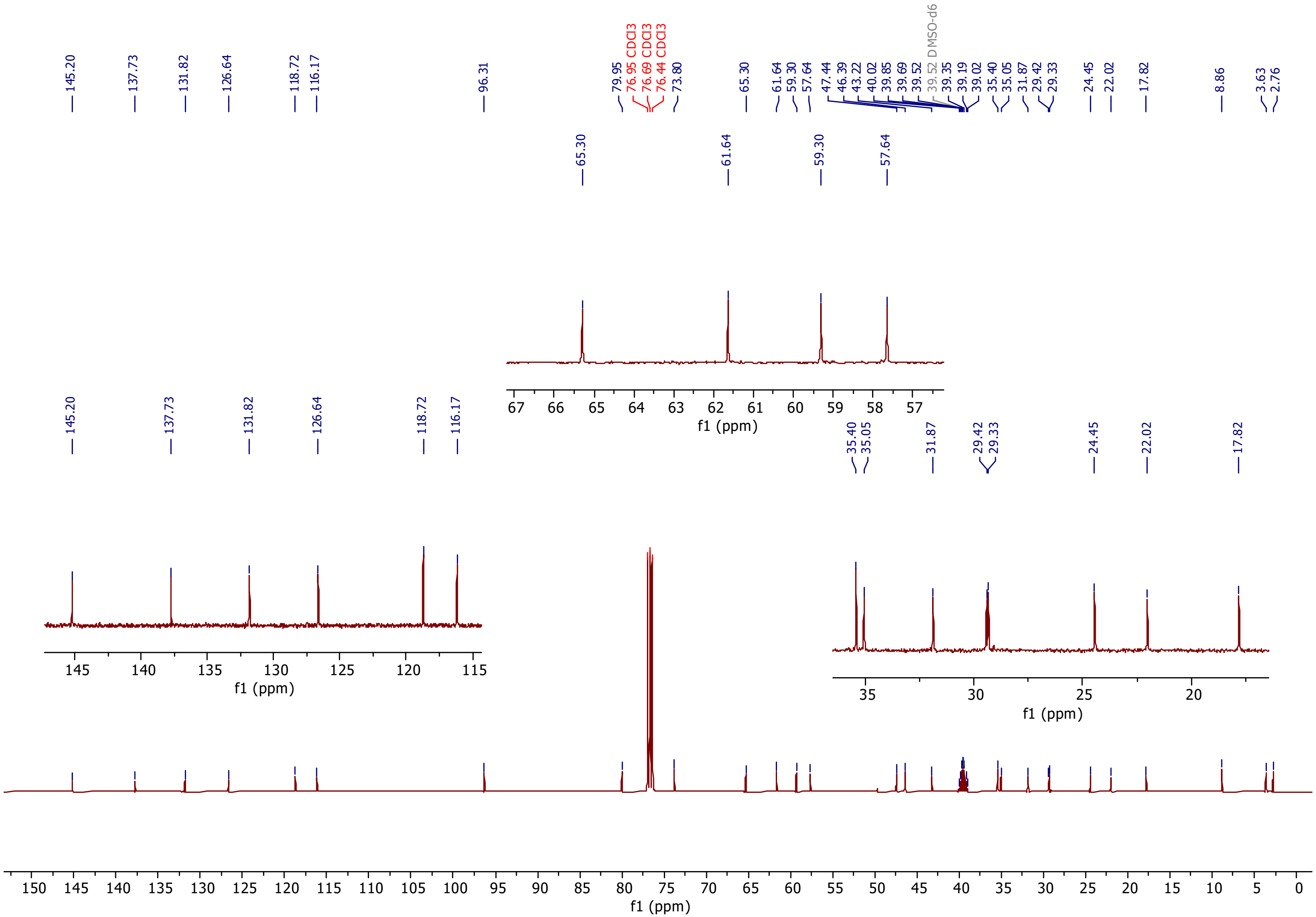

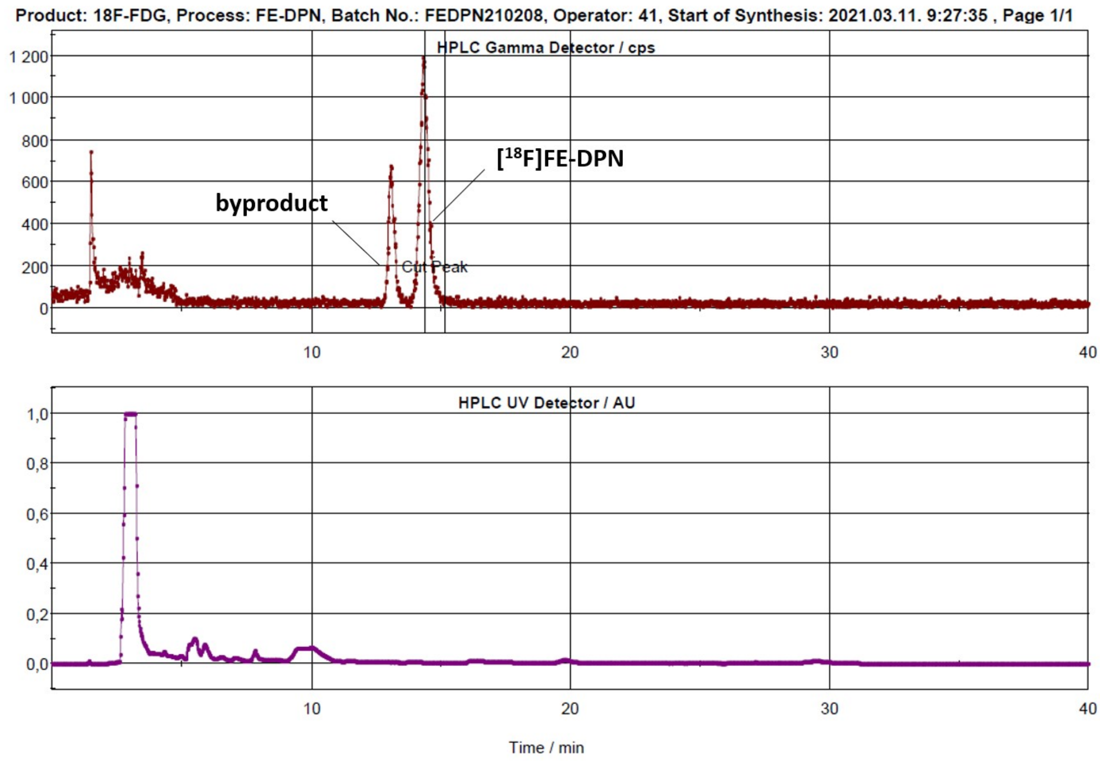

Figure S3 Semi-preparative HPLC chromatogram of the reaction mixture when **C** eluent was used as an elution mixture for [^{18}F]F$^-$ separation with Sep-Pak QMA light cartridge.

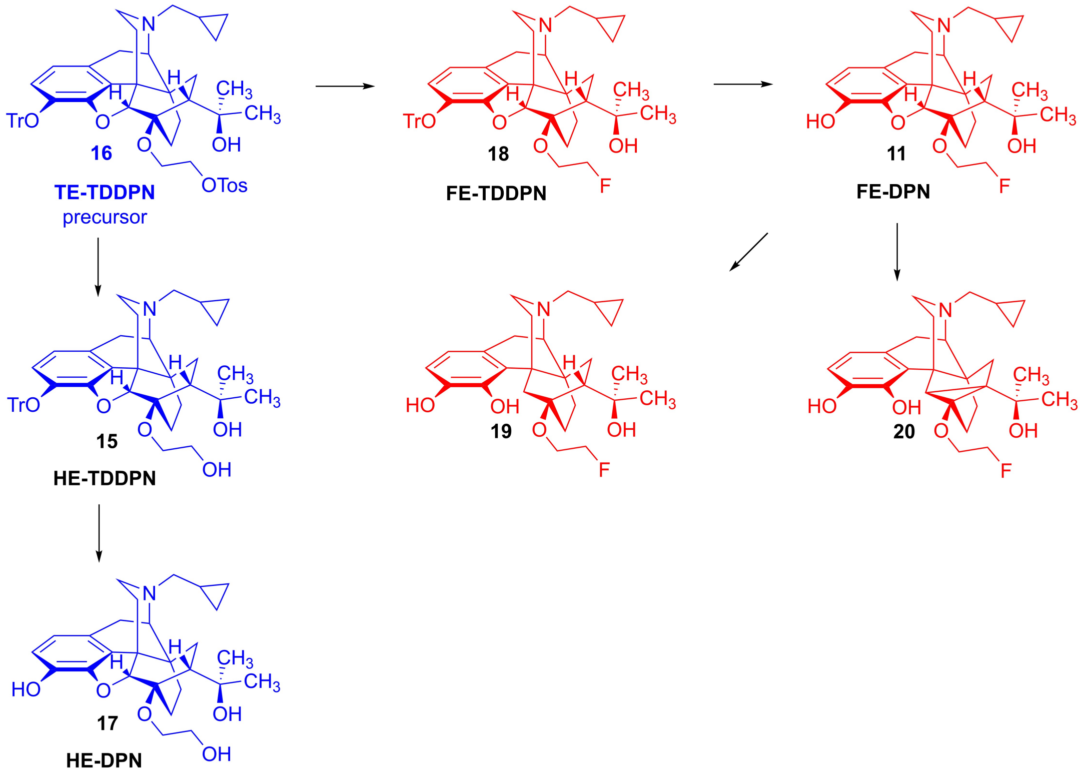

Scheme S1 Chemical structure of possible by-products

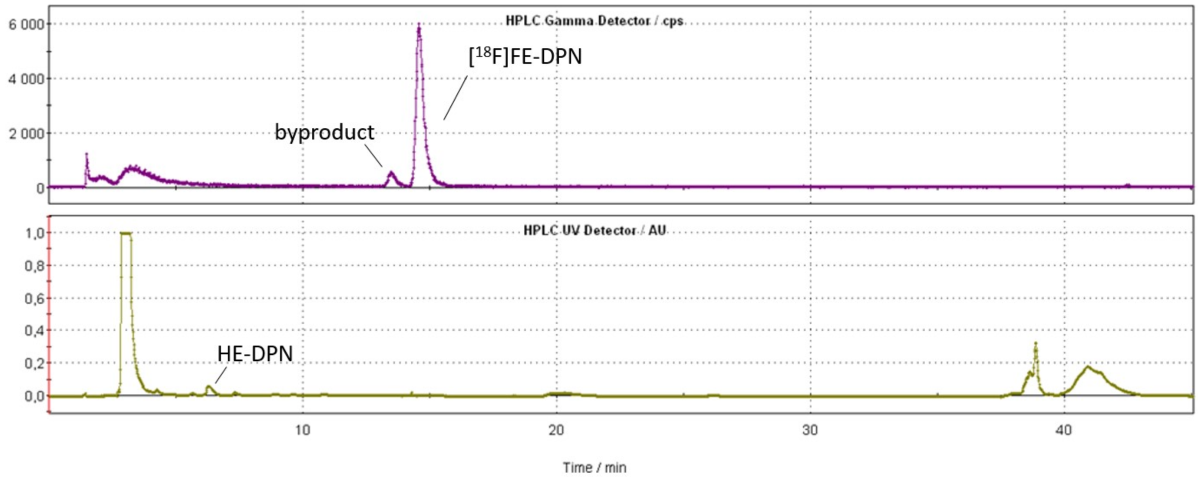

Figure S4 Semi-preparative HPLC chromatogram of the reaction mixture when **D** eluent was used as an elution mixture for [^{18}F]F$^-$ separation with Oasis Max 1cc cartridge.

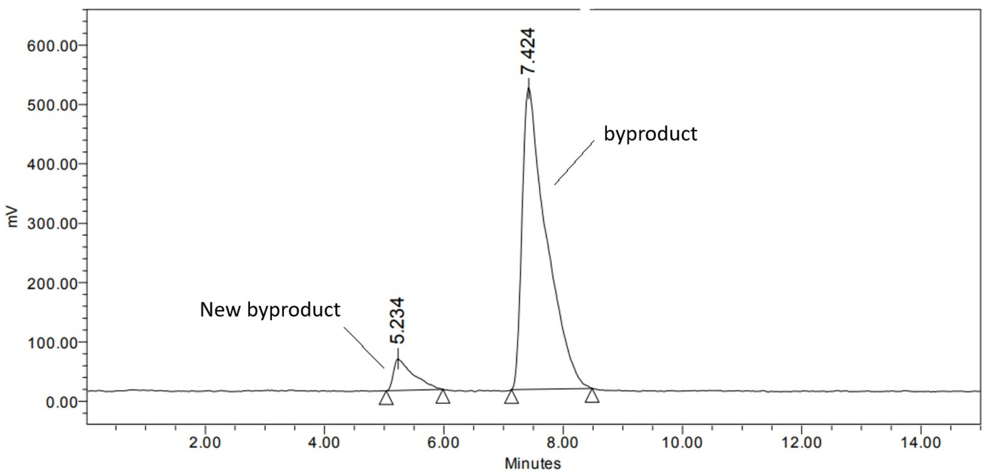

Figure S5 Analytical HPLC chromatogram of the collected by-products when **E** eluent was used as an elution mixture for [^{18}F]F$^-$ separation with Oasis Max 1cc cartridge.
